# Ongoing oxidative stress in individuals with post-acute sequelae of COVID-19

**DOI:** 10.1515/nipt-2022-0006

**Published:** 2022-08-15

**Authors:** Muhammad G. Saleh, Linda Chang, Huajun Liang, Meghann C. Ryan, Eric Cunningham, Jonathan Garner, Eleanor Wilson, Andrea R. Levine, Shyamasundaran Kottilil, Thomas Ernst

**Affiliations:** Department of Diagnostic Radiology and Nuclear Medicine, University of Maryland School of Medicine, Baltimore, MD, USA; Department of Neurology, University of Maryland School of Medicine, Baltimore, MD, USA; Department of Neurology, Johns Hopkins University School of Medicine, Baltimore, MD, USA; Department of Medicine, Division of Infectious Disease, Institute of Human Virology, University of Maryland School of Medicine, Baltimore, MD, USA; Division of Pulmonary & Critical Care Medicine, Department of Medicine, University of Maryland School of Medicine, Baltimore, MD, USA

**Keywords:** COVID-19, GABA, glutathione, SARS-CoV2

## Abstract

**Objectives:**

Coronavirus disease 2019 (COVID-19) caused by SARS-CoV-2 infection is associated with lower plasma glutathione (GSH) levels due to oxidative stress. However, plasma levels may not reflect brain GSH levels. Individuals with post-acute sequelae of COVID-19 (PASC) have a higher prevalence of cognitive fatigue, which might be related to altered brain γ-aminobutyric-acid (GABA) levels. Hence, our study aims to measure the brain GSH and GABA levels in PASC.

**Methods:**

29 PASC participants and 24 uninfected controls were recruited for this study. Each was evaluated with detailed neuropsychiatric assessments and an edited proton MRS (Hadamard Encoding and Reconstruction of Mega-Edited Spectroscopy, HERMES) method to measure GABA and GSH concentrations in predominantly grey matter (GM) and predominantly white matter (WM) brain frontal voxels.

**Results:**

PASC participants were 219 ± 137 days since their COVID-19 diagnosis. Nine individuals with PASC were hospitalized. Compared to controls, individuals with PASC had similar levels of GABA in both brain regions, but lower GSH and greater age-related GSH decline in the frontal GM region.

**Conclusions:**

The lower-than-normal frontal GM GSH level in participants with PASC suggest that they have ongoing oxidative stress in the brain, and that older individuals may be even more vulnerable to oxidative stress.

## Introduction

Severe acute respiratory syndrome coronavirus 2 (SARS-CoV-2) infection results in the coronavirus disease 2019 (COVID-19) and causes severe illness and death in a small percentage of individuals. Although the majority survive the illness, up to 30% may develop “long COVID” with persistent neuropsychiatric symptoms and cognitive fatigue – a decline in cognitive functioning during sustained mental work [1]. The mechanism for these long COVID symptoms is unknown. One possibility may be due to oxidative stress since SARS-CoV-2 infection was associated with excessive levels of plasma reactive oxygen species (ROS) and higher plasma ratios of ROS to antioxidant glutathione (GSH), which indicated oxidative stress [2, 3]. However, plasma GSH concentrations may not reflect brain GSH concentrations due to the limited uptake of GSH by the brain [4]. Furthermore, abnormalities in γ-aminobutyric acid (GABA) level was suggested in those with cognitive fatigue [5]; however, no prior study investigated brain GABA levels in COVID-19 survivors.

**Figure 1:**
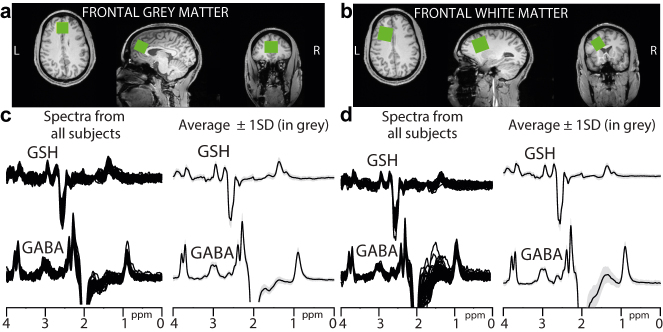
Edited MRS voxel locations (green boxes) in the (a) frontal grey matter (frontal GM) and (b) frontal white matter (frontal WM). GSH and GABA difference spectra from all participants along with their average spectrum ± 1SD (in greyscale) acquired from the (c) frontal GM and (d) frontal WM. **Participants:** 29 participants with PASC and 24 uninfected controls underwent brain MRI and HERMES scans (Table 1). The controls had no prior COVID-19 symptoms and provided a documented negative COVID-19 polymerase chain reaction (PCR) test within seven days before enrollment and a negative COVID-19 rapid antigen test on-site. PASC participants provided documented medical records for COVID-19, including the positive PCR tests for SARS-CoV-2 and whether they were hospitalized. The study protocol and consent forms were approved by the local Institutional Review Board. All participants provided written informed consent, and had a structured neuropsychiatric evaluation, detailed assessments for their COVID-19 illness, urine toxicology screening, and menstrual information in the women before the brain imaging. **Acquisition protocol:** Brain MRI and MRS scans were performed using a 3T Siemens Prisma scanner (Siemens, Erlangen, Germany) with a 64-channel head coil. In every participant, a whole brain structural scan with 1 mm^3^ isotropic resolution was acquired to guide voxel placements in the frontal grey matter (frontal GM) and frontal white matter (frontal WM, Figure 1(a) and (b)) for the HERMES sequence and unsuppressed water measurements. HERMES comprises four sub-experiments applying either a dual-lobe editing pulse to both GABA at 1.9 ppm and GSH at 4.56 ppm (A: ON_GABA_, ON_GSH_), a single-lobe editing pulse to GABA only (B: ON_GABA_), a single-lobe editing pulse to GSH only (C: ON_GSH_), or a single-lobe editing pulse at 7.5 ppm (D: OFF_GABA_, OFF_GSH_). The Hadamard combination of these sub-experiments results in GSH-edited (A–B + C–D) and GABA-edited (A + B–C–D) spectra. The remaining acquisition parameters were 25 × 30 × 25 mm^3^ (frontal GM) and 30 × 25 × 25 mm^3^ (frontal WM), TE/TR 80/2000 ms, 20 ms editing pulse duration, 4096 datapoints, 4 kHz spectral width, and 320 transients (16 transients for unsuppressed water measurements). PASC participants were also assessed for fatigue using Patient-Reported Outcomes Measurement Information System [17]. **Data processing:** Data were processed in Gannet [18, 19]. The data were combined to generate GSH- and GABA-edited spectra. The signals from GABA (GABA + macromolecules) at 3 ppm, GSH at 2.95 ppm, and the unsuppressed water at 4.68 ppm were modeled to generate absolute concentrations in institutional units. Brain structural images were segmented to calculate fractions of grey matter (GM), white matter (WM), and cerebrospinal fluid in each voxel to correct the concentrations for relaxation and partial volume effects [20]. The Cr signal at 3 ppm and the N-acetylaspartate (NAA) signal at 2 ppm were used to estimate magnetic field (*B*_0_) drift and signal linewidth at full-width half-maximum, respectively. The mean and three times the standard deviation (3SD) of GABA and GSH modeling errors and linewidth were calculated for every region. The frontal GM/WM GSH (and GABA) models had 3SD above mean error value of ∼24%/∼19% (and ∼18%/∼14%) and linewidth value of ∼14 Hz/∼14 Hz (and ∼30 Hz/∼39 Hz). These values were used as thresholds for data rejection before further analysis.

Therefore, we used proton magnetic resonance spectroscopy (^1^H-MRS) to measure GSH and GABA levels in the brain. However, GSH and GABA assessments using ^1^H-MRS are difficult at clinical (≤3T) MR scanners due to the overlap of some of their resonances with that of total creatine (tCr). Hadamard Encoding and Reconstruction of Mega-Edited Spectroscopy (HERMES) is a novel spectral editing method that selectively edits GSH and GABA simultaneously and eliminates the overlapping tCr resonance [6]. Here, we use HERMES to measure brain GSH and GABA levels from both predominantly grey (frontal GM) and predominantly white matter (frontal WM) voxels in the frontal brain region (Figure 1a and b) in individuals with post-acute sequelae of COVID-19 (PASC).

## Results

Participant characteristics are reported in Table 1. PASC and control participants had similar age, sex, racial distributions, education levels, and the indices of social position, calculated using the Hollingshead Four Factor Index of Socioeconomic Status [7]. PASC participants were 219 ± 137 days since their COVID-19 diagnosis; nine of them were hospitalized. The PASC and control groups had similar proportions with comorbid conditions prior to COVID-19; some in each group had hypertension, diabetes, depression, anxiety, or obesity. The two groups also had similar past month’s usage of tobacco (one per group), social alcohol use (83% vs. 79%), or occasional marijuana use. The most prevalent neuropsychiatric symptoms in the PASC group were difficulty with concentration (90%) and memory (76%), as well as fatigue (86%).

**Table 1:** Participant characteristics, metabolite concentrations, and tissue composition.

	Control (n=24)	PASC (n=29)	p-Value
Age (years)	44.3 ± 12.5	42.4 ± 12.3	0.57^a^
Sex (male/female^e^)	10/14	10/19	0.80^b^
Race (white/non-white)	9/15	19/10	0.08^b^
Index of social position	27.8 ± 13.6	30.4 ± 14.1	0.50^a^
Body mass index (lb/in^2^)	27.6 ± 6.7	30.7 ± 8.0	0.13^a^
**Education level**			
≤ High School/College/Graduate	2/10/12	5/16/8	0.22^b^
**COVID-19 history**			
Days since diagnosis	**–**	219.3 ± 136.7	**–**
# Hospitalized/# not hospitalized	**–**	9/20	**–**
**# With Co-morbid conditions prior to COVID-19**			
Hypertension/diabetes	1/0	6/4	0.11^c^/0.12^c^
Depression/anxiety	2/2	5/5	0.44^c^/0.44^c^
Overweight/obese	6/7	9/14	0.68^b^
Past month tobacco/alcohol/marijuana^f^	1/20/1	1/23/4	1^c^/0.98^c^/0.36^c^
**Neuropsychiatric symptoms (n, %)**			
Concentration/memory	**–**	26 (90%)/22 (76%)	**–**
Fatigue	**–**	25 (86%)	**–**
Depression and/or anxiety	**–**	20 (69%)	**–**
Insomnia/confusion	**–**	17 (59%)/20 (69%)	**–**
Myalgia	**–**	19 (66%)	**–**
Headaches/dizziness	**–**	17 (59%)/14 (48%)	**–**
Hyposmia/dysgeusia	**–**	17 (59%)/15 (52%)	**–**
**Metabolite concentrations (i.u.)**			
Frontal GM GSH	1.67 ± 0.36	1.45 ± 0.32	**0.015** ^d^
Frontal WM GSH	1.41 ± 0.38	1.58 ± 0.48	0.142^d^
Frontal GM GABA	3.24 ± 0.65	3.38 ± 0.84	0.499^d^
Men	2.88 ± 0.58	3.08 ± 0.87	0.556^a^
Women	3.47 ± 0.59	3.54 ± 0.81	0.785^a^
Frontal WM GABA	3.05 ± 0.37	3.13 ± 0.48	0.634^d^
Men	3.14 ± 0.31	2.98 ± 0.53	0.429^a^
Women	2.99 ± 0.40	3.21 ± 0.44	0.151^a^
**Frontal GM tissue composition (%)**			
GM	53.9 ± 1.0	55.2 ± 0.8	0.29^a^
WM	32.6 ± 1.1	30.3 ± 1.1	0.15^a^
**Frontal WM tissue composition (%)**			
GM	26.0 ± 0.8	25.8 ± 0.6	0.87^a^
WM	71.2 ± 1.1	71.6 ± 0.7	0.81^a^

Data are presented as Mean ± SD or n or %. ^a^t-test (parametric or non-parametric), ^b^Chi-Square test, ^c^Fisher’s Exact Test, ^d^ANCOVA; PASC=participants with post-acute sequelae of COVID-19; GM=Grey Matter; WM= White Matter; i.u.: institutional units; GSH: glutathione; GABA: γ-aminobutyric acid. ^e^Among 33 women, four did not reveal their day-of-cycle information, 11 were in the follicular phase of the menstrual cycle (day one to 15), and 18 were in the non-follicular phase of the cycle. Women in the non-follicular phase included 6 in the luteal phase (day 16–45), three were on contraceptives and did not have a normal menstrual cycle, 4 had hysterectomy/oophorectomy, and 5 were postmenopausal. ^f^Includes 2 PASC who used cannabidiol. All had negative urine toxicology screenings.

GSH and GABA difference spectra from all participants in both regions are shown in Figure 1(c) and (d). B_0_ drift from the 11-min acquisition in the frontal GM was 0.35 ± 1.68 Hz and frontal WM was 1.34 ± 1.59 Hz, whereas the linewidth was 8.95 ± 0.97 Hz in the frontal GM and 7.83 ± 0.88 Hz in the frontal WM, suggesting excellent frequency stability and B_0_ homogeneity during acquisition. Two GSH and three GABA modeled spectra were removed due to either modeling errors or linewidths exceeding the thresholds.

**Figure 2:**
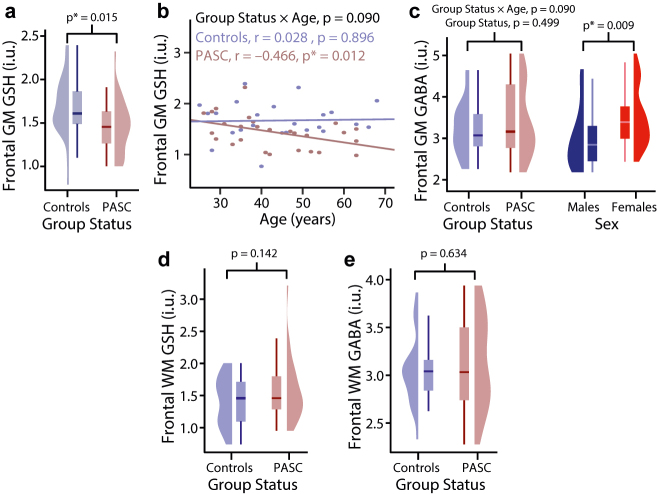
(a) The frontal GM GSH is significantly lower in PASC compared to controls; (b) the same region shows age-related GSH decline in PASC; (c) the frontal GM GABA levels show a trend for interaction between group status and age, but non-significant age-related changes between the two groups (controls: r=−0.31, p=0.15; PASC: r=0.21, p=0.27); the frontal GM GABA is significantly lower in men than in women; (d) frontal WM GSH and (e) frontal WM GABA are not significantly different between the two groups. frontal GM: frontal grey matter, frontal WM: frontal white matter, PASC: participants with post-acute sequelae SARS-CoV-2 infection, i.u.: institutional units. **Analyses:** Statistical analyses were conducted in the R software (http://www.r-project.org). Two-way analyses of covariance (ANCOVA) were performed for both brain regions to evaluate the effects of group status (PASC or controls), age and sex as covariates, and the interactions of group-by-age and group-by-sex on GABA and GSH concentrations separately. If an interaction was significant or showed a trend (p≤0.1), post-hoc correlation analyses were performed using Pearson or Spearman correlations (depending on the residual distribution); otherwise, interaction terms were removed from the model. Frontal GM and WM GABA and GSH levels (separately) between hospitalized and non-hospitalized PASC participants were examined. Correlations between frontal GM and WM GABA and GSH levels (separately) and time since infection were performed. Furthermore, GABA levels within women (irrespective of status) were assessed using a Student’s *t*-test between the follicular and non-follicular phase. p-Values ≤0.05 were considered significant. Values are presented as mean ± SD.

PASC participants and controls had similar tissue composition of MRS voxels in both brain regions. The tissue corrected GSH and GABA concentrations are reported in Table 1. GABA and GSH (separately) from both regions did not correlate with SES. In the frontal GM, GSH concentrations were significantly lower in PASC (Figure 2(a): −13%, p=0.015) than in uninfected participants. Group status and age showed a trend for interaction for GSH in the frontal GM (Figure 2(b): interaction-p=0.090), since only the older PASC individuals had lower GSH (r=−0.466, p=0.012), but not the controls. Group status and age also showed a trend for interaction for the frontal GM GABA (p=0.087), driven by opposite (although non-significant) age-related changes between the two groups (Controls: r=−0.31, p=0.15; PASC: r=0.21, p=0.27). In addition, fatigue scores in participants with PASC did not correlate with the frontal GM GABA (p=0.28) and WM (p=0.13) levels. Furthermore, regardless of PASC status, the frontal GM GABA levels were lower in men than in women (Figure 2(c): −17.6%, p=0.009). However, in the frontal WM, neither GSH nor GABA showed significant group differences (Figure 2d and e). GABA levels of women in both brain regions were not significantly different between the follicular and non-follicular phase. Our analyses also yielded non-significant differences in the frontal GM and WM GSH concentrations between sexes. Hospitalized and non-hospitalized participants revealed non-significant differences in GABA and GSH concentrations in both regions. There was no association between GSH and GABA levels (separately) from both regions with time since infection.

## Discussion

We evaluated brain abnormalities in PASC using an edited MRS technique to measure brain GSH and GABA concentrations non-invasively. Our PASC participants showed lower than normal GSH levels in the frontal GM approximately seven months after their acute illness. The reduced brain GSH levels suggest ongoing oxidative stress in individuals with PASC. In contrast, our PASC participants had relatively normal GABA levels in both brain regions.

Lower brain GSH levels in PASC participants is consistent with the reduced plasma GSH levels and elevated ROS biomarkers reported in COVID-19 patients [3]. Elevated ROS could result from SARS-CoV-2 binding with the functional receptor angiotensin-converting enzyme-2 (ACE2) to gain entry into a cell [8]. Upon binding, ACE2 overstimulates ROS generation and increases oxidative stress [8]. Higher levels of ROS may impair adaptive immunity, increase vulnerability to viral infections, induce oxidative damage of endogenous molecules, leading to cell damage [2]. Since higher levels of the antioxidant GSH are essential for reducing ROS, the administration of N-acetylcysteine (NAC) [9], which is a cysteine precursor for GSH biosynthesis, might be useful for enhancing GSH levels in individuals with PASC [10].

The lower brain GSH levels was primarily in older post-COVID-19 individuals, suggesting that age, a known risk factor for lower antioxidant capacity and higher oxidative stress [2, 3], may exacerbate or contribute to the effects of COVID-19 in the brain. The age-related decline in GSH is similar to the age-related decline in plasma GSH levels in older COVID-19 patients [11]. Since older COVID-19 patients tend to develop a more severe illness, deficient GSH may have negative impacts to the health of older individuals with PASC [12, 13].

Although the GABA levels were not different between the post COVID-19 and control groups, the frontal GM GABA levels were higher in women than in men, which are consistent with some but not all prior reports [14, 15], and the reasons are not fully understood. Neuroactive steroids, including progesterone and testosterone, have complex modulatory effects on GABA release, which may vary by brain region [16]. The observed effect of sex on GABA levels is likely related to such neuroactive steroidal-related modulation of GABA. Further studies including hormonal measurements are needed to evaluate their effects on GABA levels in different brain regions.

The present study has some limitations. First, we did not perform antibody tests in the controls to ensure they did not have past asymptomatic infections. Those tests were not as readily available but should be done in future studies. Second, we only evaluated two brain regions, but other brain regions might also be affected. Future studies may consider using MRS methods that can investigate multiple regions simultaneously. Third, the GABA signal also contains signals from macromolecules and homocarnosine, future studies applying macromolecular suppressed editing are required to address this issue. Lastly, our sample size is modest, which might have led to borderline significance for some measures (e.g., the group-by-age interaction for GSH or for GABA in the frontal GM). A larger sample size including more older participants is needed to further evaluate whether age-related decline in GSH or GABA occurs in recovered COVID-19 participants.

## Conclusions

Lower GSH levels in the frontal GM region of recovered COVID-19 patients with persistent neuropsychiatric symptoms suggest ongoing oxidative stress.
